# Tuberculosis of the Oral Cavity Affecting Alveolus: A Case Report

**DOI:** 10.1155/2011/945159

**Published:** 2011-06-30

**Authors:** Bipin Kumar

**Affiliations:** ^1^Department of Pathology, B. P. Koirala Institute of Health Sciences, Dharan, Nepal; ^2^Department of Pathology, Indira Gandhi Medical College and Research Institute, Kathirkamam, Puducherry 605009, India

## Abstract

We report a first case of tuberculosis of oral cavity affecting the left alveolus from Nepal in a 63-year-old male who came to otorhinolaryngology outpatient department with a complaint of an ulcer in the oral cavity and pain in bilateral ear and throat. An ulcer measuring 1.8 cm in diameter with irregular friable margin and bleeding on touch was found in the left upper alveolar region of the oral cavity. Biopsy from the ulcer margin revealed histological features of necrotizing granulomatous lesion. Stain for acid fast bacilli was positive.

## 1. Introduction

Tuberculosis is an infectious granulomatous disease caused by mycobacterium [1–3]. The lung is the most common site of involvement, and it rarely involves the oral cavity [1, 2]. Most of the tuberculous lesions of oral cavity are secondary to lung disease, usually seen in elderly patients [1]. Primary tuberculosis of the oral cavity is rare and is most commonly found in children and adolescents rather than in adults [1, 2]. Here we report a first case of tuberculosis of oral cavity affecting the left alveolus in a 63-year-old male from Nepal.

## 2. Case Report

**Figure 1:**
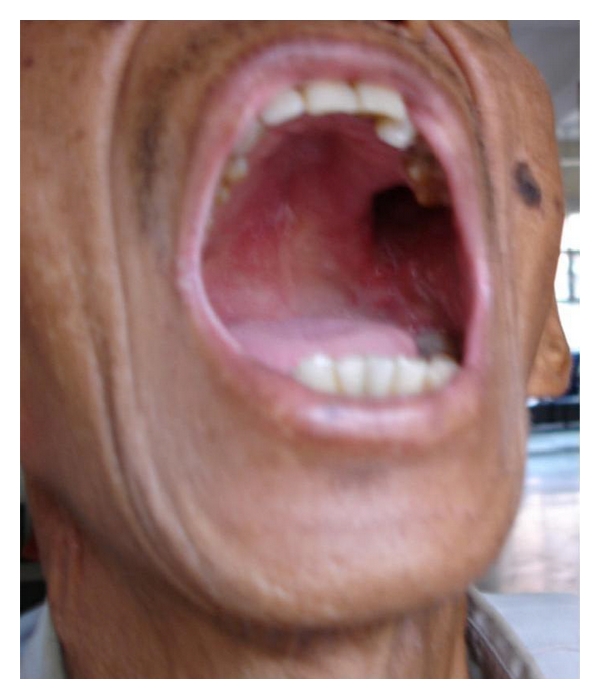
Ulcer in oral cavity affecting upper left alveolus.

A 63-year-old male farmer of a suburban area came to the otorhinolaryngology outpatient department of our hospital with complaints of a painless ulcer in the oral cavity for 10 days and pain in both the ears and throat for 3 days. On local examination, a painless ulcer measuring 1.8 cm in greatest diameter with irregular, friable margin and bleeding on touch was found affecting the left alveolar mucosa, involving premolar and molar region and extending laterally into the gingivolabial sulcus and medially into the soft palate (Figure 1). Only the incisors, canine, and the first premolar teeth were intact on the affected side, and the throat was congested. A clinical diagnosis of cancer of the left alveolus in the oral cavity was made; an incisional biopsy was taken from the margin of the ulcer and sent for histopathological examination. Routine hematological and biochemical investigations and the chest X-ray did not reveal any abnormality. No radiographic evidence of involvement of underlying bone was seen. Serological tests done for syphilis and HIV were found negative.

**Figure 2:**
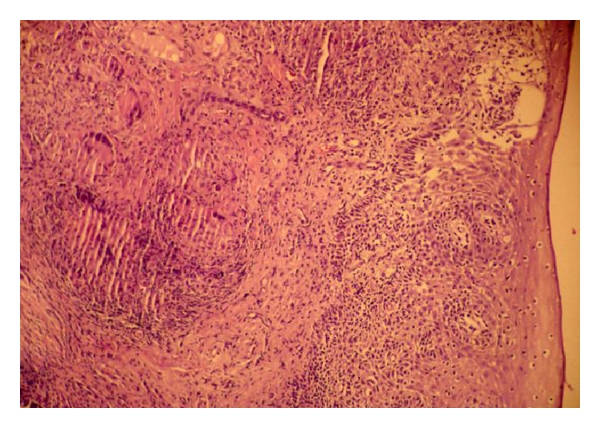
Section showing epithelioid cell granuloma with Langhans' type of giant cells. (H & E; X10).

**Figure 3:**
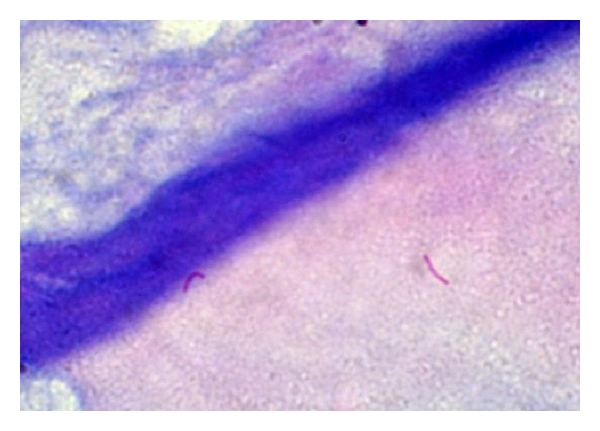
Z-N staining showing acid fast bacilli (X100; in oil immersion lens).

Microscopic examination showed stratified squamous epithelium and subepithelium revealing crushing artifacts with presence of multiple necrotizing epithelioid cell granuloma and Langhans' type of giant cell (Figure 2). Ziehl-Neelsen stain of the tissue showed many acid fast bacilli (Figure 3). Malignancy was not seen in serial section examined. Silver stain done on the section did not show spirochetes or fungi. On the basis of above findings, a diagnosis of tuberculosis of left upper alveolus was made. Subsequent history taken after the diagnosis, revealed history of exodontias, one month back of the presentation of an ulcer and occasional use of raw milk. There was no family history of tuberculosis.

The patient was treated with multidrug antitubercular regimen. The drug given for initial two months were isoniazid 400 mg, rifampicin 600 mg, ethambutol 750 mg, and streptomycin 1000 mg. The ulcerative lesion was completely healed after 2 months of therapy. The drugs isoniazid 400 mg and rifampicin 600 mg were continued for further four months. No recurrence was observed in the follow-up period of a two-year duration after the completion of full-course multidrug regimen.

## 3. Discussion

Tuberculosis of oral cavity is a rare lesion [1]. It may be primary or secondary to pulmonary tuberculosis [1]. Secondary tuberculosis is more common in case of oral tuberculosis and radiographic evidence of pulmonary involvement could be demonstrated in 93.3% of cases [3]. The primary tuberculosis of oral cavity is unusual, and most references in medical literature are case reports [4–11]. The intact oral mucosa is relatively resistant to invasion of bacilli and this only happens in 0.05–0.1% of cases with tuberculosis [4, 5]. The resistance is caused by the cleansing action of saliva, the presence of salivary enzymes, tissue antibodies, oral saprophytes, and thickness of the protective epithelial covering [1, 6]. Micro-organism needs a disruption of oral mucosa to become pathogenic [7]. Any break or loss of the natural barrier, which may result from trauma, inflammatory conditions, tooth extraction, or poor oral hygiene, may provide a route of entry for the mycobacterium [1, 6, 8]. The most common sites for oral tuberculosis are tongue, gum, and palate [1, 4, 9]. Other sites include the lip, cheek, uvula, and alveolar mucosa [5, 8]. The lesion of oral cavity may accompany lesions of the pharynx, larynx, lung, and lymph nodes [10]. History of exodontias and history of consumption of unboiled raw milk were also found [1, 7]. Oral tuberculosis in HIV-infected patients was also reported [9].

 In the present case, ulcerative lesion in left upper alveolar mucosa was found after one month of teeth extraction. The patient gave the subsequent history of occasional use of raw milk after the histopathological diagnosis of oral tuberculosis and chest X-ray did not reveal any lesion. Hence, in our opinion, the lesion in present case is primary and it is caused by mycobacterium bovis infection. Similar case report affecting the tongue is well documented [11]. The primary tuberculous oral lesion was more commonly found in children and adolescent than in adults [1, 2]. However, cases of primary tuberculosis in adult and middle-aged persons were also reported [7]. Males were affected more than females [7]. Clinical features included oral ulcer [1, 2, 4, 9], swelling of gingival [8], mass in oral mucosa [2], odynophagia [3], fever [3, 8], weight loss [8], and lymphadenopathy [10]. Ulcer used to be single or multiple and either painful or painless [1, 2]. In the present case, the clinical features of painless ulcer with friable, irregular margin, bleeding on touch along with otalgia and painful congestion of the throat were present. Otalgia and throat pain were due to concomitant viral pharyngitis.

Differential diagnosis included malignancy, traumatic or aphthous ulcer, syphilis, sarcoidosis, and deep mycotic infections [1, 2]. The present case roused high clinical suspicion for malignancy.

Investigations done were complete blood count, sputum examination for acid fast bacilli, culture of sputum, incisional biopsy, culture of tissue, Mantoux test, polymerase chain reaction, and chest X-ray [1, 2].

In the present case aphthous ulcer was excluded by the absence of initial multiple painful lesions. Syphilitic ulcer was ruled out by serology and silver stain done on tissue section. HIV was ruled out by serology. Sarcoidosis was ruled out by the absence of lung involvement on radiological examination and presence of caseation and AFB on histopathological examination. 

Diagnosis of oral tuberculosis was based on histopathological examination and demonstration of acid fast bacilli on Ziehl-Neelsen staining [1, 8]. Due to selective scarcity of bacilli within tissue, mycobacteria can be demonstrated only in 27–60% of cases [2]. Culture of mycobacteria had good result but it lacks sensitivity and takes 4–6 weeks [1, 2]. Chest X-ray is done to exclude the possibility of pulmonary tuberculosis. 

All the reported cases of oral tuberculosis including the present case responded well to antitubercular drug regimen.

## 4. Conclusion

In cases of ulceroinflammatory lesion of oral cavity, tuberculosis should be considered as a differential diagnosis and incisional biopsy should be done in order to reach an accurate diagnosis. X-ray chest should also be done in each case for decision on the issue of primary versus secondary tuberculosis of oral cavity.
